# 

**Published:** 1883-07

**Authors:** 


PAGE

ORIGINAL COMMUNICATIONS.—Actinomykosis ... 183
Some Diseases of the Eye in Lower Animals. William Oliver Moore, M.D. ... 199
Abortion. A. S. Heath, M.D. ... 204
Physical Diagnosis. E. Benjamin Ramsdell, M.D. ... 205
Supra-Pubic Cystotomy. Joseph W. Stickler, M.D. ... 213
Lameness. E. A. McLellan, D.V.S. ... 215
EDITORIAL.—The Germ Theory—Columbia Veterinary College—Mr. Sander's Mission ... 225-230
REPORT OF COMMENCEMENTS AND SOCIETIES ... 232
CORRESPONDENCE ... 254
CASE DEPARTMENT.—Strychnine Poisoning in a Dog—Ingrowing Eyelashes—Bursal Enlargement of the Tendon of the Flexor Pedis Perforans—Removing a Foreign Body from the Œsophagus—Abortion Epizootic Cellulitis—Incipient Stages of Scabies from Sarcoptes Canis—Spinal Meningitis—A Case of Jaundice—Multiple Comminuted Fracture of the Sesamoid Bones of both Forward Extremities—Keratitis in a Dog—Intestinal Calculus from a Horse—Hydatids of the Liver from a Camel—Pleuro-Pneumonia Contagiosa—Chorea in a Horse ... 235-245
REVIEWS.—Feeding Animals—Book and Pamphlets Received ... 245-
OBITUARY ... 247
PROGRESS OF VETERINARY SCIENCE.—A Case of Transmission of Tubercle from the Human Species to the Domestic Cat—Tuberculosis and Hydrothorax in a Female Rhinoceros—Scarlatina Among Horses—Anthrax in the Hog Somewhat Different from the Common Form—Immunity of Animals from Syphilitic Inoculation—The Reparative Process in Cartilage—Examination of a Hybernating Snake—Consumption and Tuberculosis ... 249-253
NEWS AND MISCELLANY.—Parasites in American Pork—Presentation—Prof. Virchow's Health—Red-water in Cattle—Abnormal Periods of Gestation—Prof. Robertson—Personal—Horses for Food—Air-space in Cow-sheds—Cruelty to Animals—More Veterinarians Needed—M. Pasteur—A Misnomer Explained—Veterinary Hospital—Illinois, State Veterinary Convention—The Degree of LL.D.—Presentation—Glanders—The Treatment of Imported Cattle—Dr. L. Roth—Trichinæ—Cerebral Congestion—Cerebral Tumor—Improvement upon the Rary System. 254-261

